# Correlation of Ultrasound Shear Wave Elastography with Pathological Analysis in a Xenografic Tumour Model

**DOI:** 10.1038/s41598-017-00144-5

**Published:** 2017-03-13

**Authors:** Eli Elyas, Efthymia Papaevangelou, Erwin J. Alles, Janine T. Erler, Thomas R. Cox, Simon P. Robinson, Jeffrey C. Bamber

**Affiliations:** 1 CRUK and EPSRC Imaging Centre, Division of Radiotherapy and Imaging, Institute of Cancer Research, Sutton, Surrey, UK; 2 Joint Department of Physics, Institute of Cancer Research and Royal Marsden NHS Foundation Trust, Sutton, Surrey, UK; 3 Biotech Research & Innovation Centre (BRIC), University of Copenhagen, Copenhagen, Denmark; 4 The Garvan Institute of Medical Research and The Kinghorn Cancer Centre, Cancer Division, St Vincent’s Clinical School, Faculty of Medicine, University of New South Wales, Sydney, Australia; 5 Department of Clinical and Experimental Medicine (IKE), Linköping University, Linköping, Sweden; 6 MRC Centre for Transplantation, Division of Transplantation Immunology and Mucosal Biology, Guys Hospital, King’s College London, London, UK; 7 Department of Medical Physics and Biomedical Engineering, University College London, London, UK

## Abstract

The objective of this study was to evaluate the potential value of ultrasound (US) shear wave elastography (SWE) in assessing the relative change in elastic modulus in colorectal adenocarcinoma xenograft models *in vivo* and investigate any correlation with histological analysis. We sought to test whether non-invasive evaluation of tissue stiffness is indicative of pathological tumour changes and can be used to monitor therapeutic efficacy. US-SWE was performed in tumour xenografts in 15 NCr nude immunodeficient mice, which were treated with either the cytotoxic drug, Irinotecan, or saline as control. Ten tumours were imaged 48 hours post-treatment and five tumours were imaged for up to five times after treatment. All tumours were harvested for histological analysis and comparison with elasticity measurements. Elastic (Young's) modulus prior to treatment was correlated with tumour volume (r = 0.37, p = 0.008). Irinotecan administration caused significant delay in the tumour growth (p = 0.02) when compared to control, but no significant difference in elastic modulus was detected. Histological analysis revealed a significant correlation between tumour necrosis and elastic modulus (r = −0.73, p = 0.026). SWE measurement provided complimentary information to other imaging modalities and could indicate potential changes in the mechanical properties of tumours, which in turn could be related to the stages of tumour development.

## Introduction

Elastography, the imaging of elastic properties of soft tissues, is a range of imaging techniques that provide useful information about various pathological processes such as cancer, fibrosis and atherosclerotic plaques. The importance of clinical elasticity imaging lies in its ability to provide complementary information to other imaging techniques. Elastography can detect lesions, invisible or barely visible by ultrasound (US) and Magnetic Resonance Imaging (MRI)^1, 2^. In addition, it has been used to detect treatment response to drugs in tumour models^3^ and in patients^4^, especially in oncology, as the change in the elastic ‘phenotype’ is considered a manifestation of mechanical processes determined on a cellular level such as desmoplastic stroma and inflammation which may precede macroscopic changes^5^.

Until recently, rheometry^6^, mechanical force probing^7^ or Atomic Force Microscopy^8^ were the main methods to assess tissue elasticity in biopsy samples. However these methods, which cannot be performed in real time, require the acquisition of an invasive biopsy, and only account for a small part of the whole tissue (the biopsy site). This is clinically relevant given the high degree of heterogeneity in solid tumours. Removing tissue from its original environment might also cause alterations in its mechanical properties which affect the obtained results.

Although ultrasound has always been a major tool for elastography application, an *in vivo* quantitative application has only recently emerged. The supersonic shear wave imaging (SSI) is a quantitative ultrasound imaging method that allows assessing the tissue elasticity *in vivo* ^9^. Recently, Chamming’s *et al.* used this method to successfully assess the stiffness of tumours formed from human breast cancer cells implanted first into the murine fat pad^10^ and then subcutaneously^11^. Using MR elastography (MRE) Jugé *et al.* reported a correlation between stiffness, vessel density and cellularity of colon cancer tumours subcutaneously implanted in mice^12^. In another MRE study, Li *et al.* assessed the stiffness of tumours before and after treatment with a vascular disrupting agent^3^.

However, all these studies used anti-angiogenic or vascular disrupting drugs. In this pilot study our purpose was to evaluate the potential of SWE to monitor the changes in tumour elastic properties as a response to cytotoxic drugs, and correlate this with histological analysis to investigate the longitudinal dependence between necrosis and elastic modulus.

## Materials and Methods

### Animal model

All procedures involving animals were performed in accordance with the local ethical review panel, the UK Home Office Animals (Scientific Procedures) Act 1986, the United Kingdom National Cancer Research Institute guidelines for the welfare of animals in cancer research^13^ and the ARRIVE guidelines^14^. All experimental protocols in the license were approved by the UK Home Office, and the individual protocols were prepared according to the licensed experimental protocols. 5 × 10^6^ SW620 human colorectal adenocarcinoma cells (European Collection of Cell Cultures, Centre of Applied Microbiology and Research, Salisbury, UK) in 100 µl of serum free culture medium were injected subcutaneously into the right flank of 15 seven-week-old female NCr nude immunodeficient mice (Charles River Ltd. UK). After the tumour was palpable and measured 2 mm by 2 mm, its volume was measured by callipers and calculated using the ellipsoid volume formula, V = π/6 × L × W × H, where L, W and H correspond to length, width and height of the tumour respectively^15^. To estimate the error, each tumour measurement was performed eight times. The potential bias was removed by making the measurer blind to the records of the calliper.

The mice were scanned by a commercial US scanner with an integrated SWE measurement property, Aixplorer (SuperSonic Imagine, Aix-en-Provence, France), every second day from day 10 after implantation, and were treated after the third scan.

The design of experiments was such to determine the optimal analysis which could be performed thereby reducing and optimising the number and size of groups using observations from previously published work. Overall, the aim was to perform experiments where a measurable effect could be determined using a minimal number of animals. Using 80% power and 95% confidence, a predicted 25% practical difference and 15% coefficient of variation, group sizes of 5 animals are sufficient, where 1 tumour was implanted per animal.

**Figure 1:**
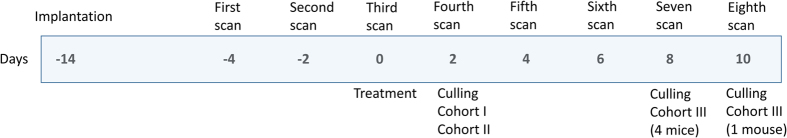
Timeline of the experiment.

The mice were randomly divided into three cohorts. Cohort I (four mice) was treated with saline solution (control); cohort II (six mice) and cohort III (five mice) were treated intraperitoneally with a single dose (100 mg/kg) of the topoisomerase inhibitor Irinotecan^16^. Mice in cohort I and cohort II were culled by cervical dislocation after the fourth scan, 48 h after the treatment. We hypothesized that drug-treated tumours would become more necrotic with time. Thus, to examine longitudinal dependence between necrosis and elastic modulus, mice in cohort III were left until their tumours reached ethical limits (six to ten days after drug treatment). Figure 1 shows a schematic timeline of the experiment.

### US-SWE

**Figure 2:**
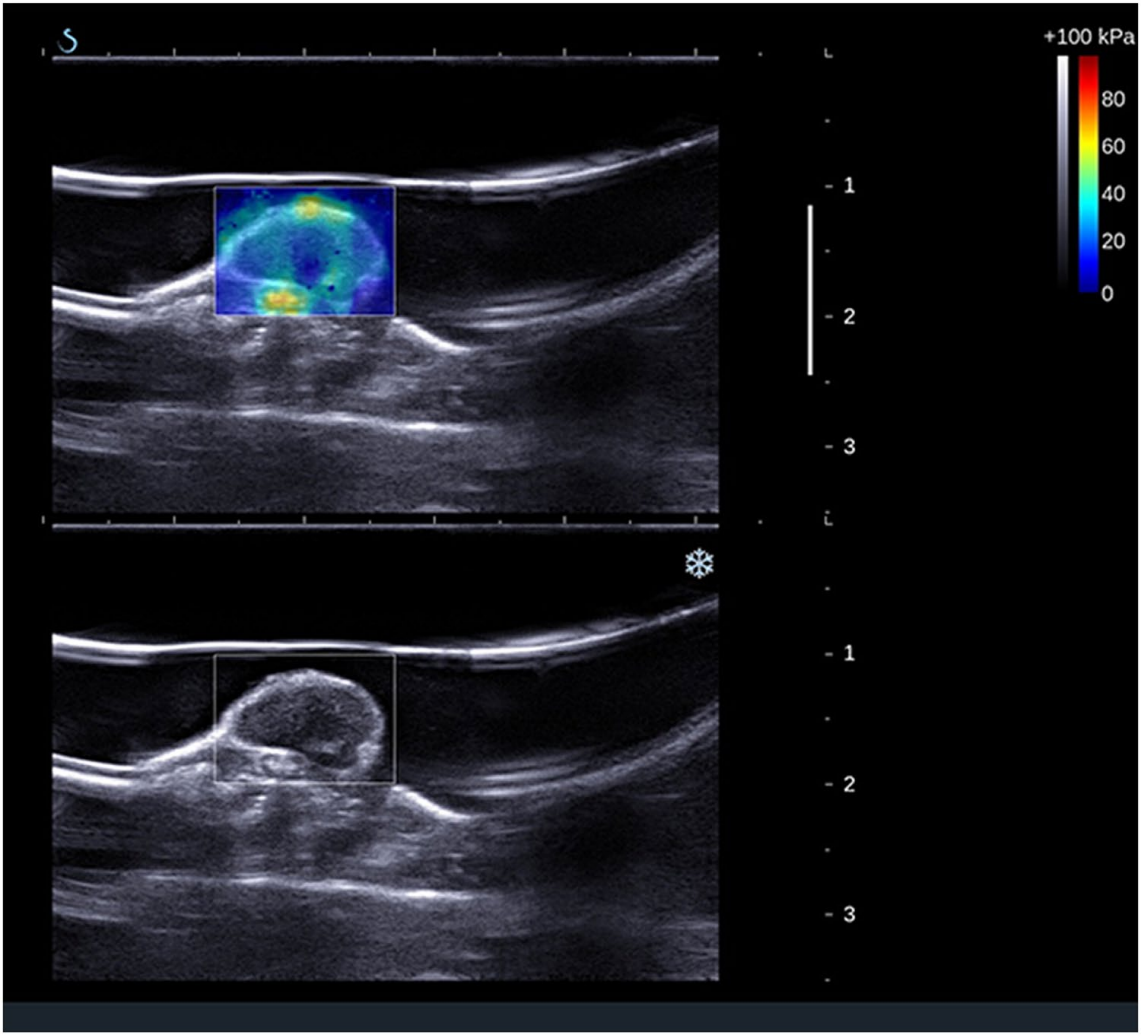
Image acquisition. B-mode image in sagittal plane of the tumour located on the flank of the mouse with (top panel) and without (bottom panel) the elasticity box. The scale on the right represents depth (cm) and the white line indicates the focus position. The colourmap indicates elastic (Young’s) modulus values in kPa. White line above the tumour is a nylon pocket.

During image acquisition, mice were placed on a table  heated to 37 °C to maintain animal core temperature under isoflurane anaesthesia (2 ml/min in 100% O_2_) for approximately 20 minutes. The US probe (SuperLinear^TM^ SL15-4, SuperSonic Imagine, Aix-en-Provence, France) was mounted on a 3D scanning motor to allow tumour positioning and eliminate artefacts during acquisition. The US probe was partially immersed in a nylon pocket water bath and degassed US gel (Skintact®, Leonhard Lang GmbH, Innsbruck, Austria) was applied to the scanning area for better coupling. Two B-mode and elasticity images, one in the transverse and one in the sagittal plane, at the centre of each tumour were obtained in each acquisition. A total of 144 ultrasound shear wave elastography measurements were performed. An example of an image is shown in Fig. 2.

**Figure 3:**
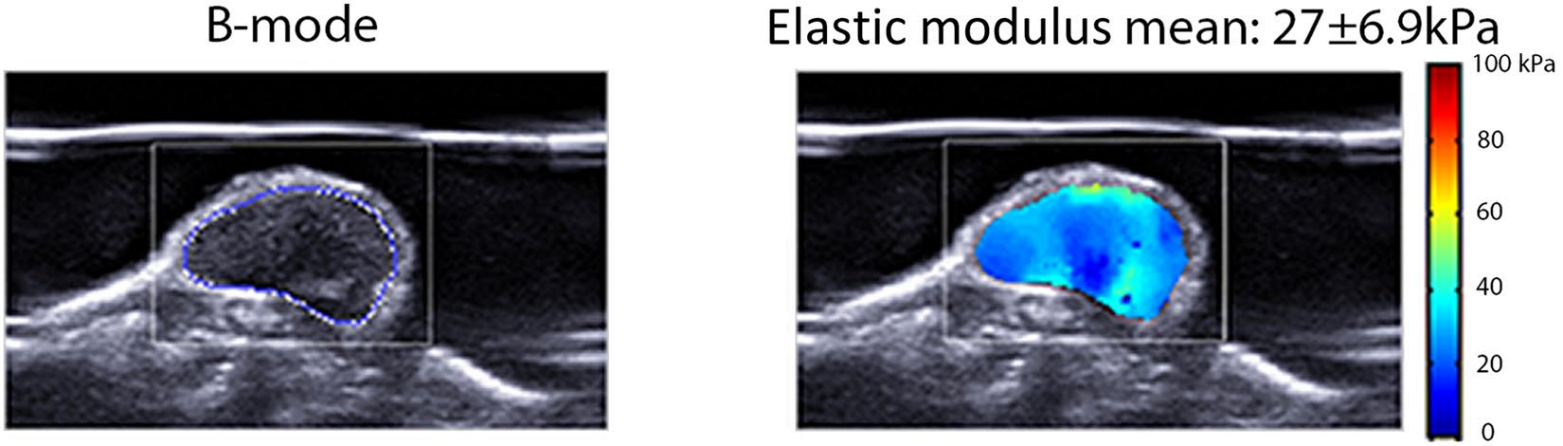
Elastic (Young’s) modulus calculation. Specially written software allows the ROI to be drawn on the B-mode (left), which is reciprocated on the elasticity image (right). The stiffness calculation was performed off-line once scan data had been collected.

An in-house program was written in MATLAB® (The MathWorks, Natick, MA, USA) to allow free hand drawing of the region of interest (ROI) and therefore produced the result only within a chosen area. The program defines the colour scale, scans the image and converts it to kPa using colourbar. Mean values of elastic modulus and standard deviation were recorded. An ROI including the whole of each tumour and excluding skin was drawn, and the mean value of the elastic modulus from sagittal and transverse planes was calculated (Fig. 3).

### Histological analysis

Tumours were excised at the end of the experiments. The tumours were fixed in 10% formalin solution (Sigma-Aldrich Chemie GmbH, Steinheim, Germany) for 24 hours and then into 70% alcohol solution where they were stored until being embedded into paraffin. The paraffin embedding was performed using a Leica EG 1160 embedder (Germany). Formalin fixed paraffin embedded tumours were sectioned using a microtome into 4 µm sections and stained with haematoxylin and eosin (H&E) for cellular visualisation, the endothelial cell marker CD31 and the connective tissue marker Masson’s trichrome. Stained sections were visualised using Cell^P (software imaging system, Münster, Germany) installed on an Olympus Bx51 microscope (Olympus Optical, London, UK) with an attached ProScan II motorized scanning stage system (Prior Scientific Instruments Ltd., Cambridge, UK). The percentage of stained area for CD31 and Masson’s trichrome included the whole area of the tumour was calculated using an in-house written Matlab script which detects the pre-defined colour in the image. With CD31 staining, the vascular areas appear in brown on a purple background, while collagen appears blue or green on the red-purple background. The script identifies brown (for CD31) or green (for Masson’s) pixels and gives their percentage. A surface area in viable regions was calculated from the H&E stained sections using ImageJ^17^.

### Statistical analysis

Two-way ANOVA was used to compare volume and elastic modulus changes between control and drug-treated groups. The p-values less than 5% were taken to be statistically significant. Pearson’s correlation was used to compare between tumour volume and tumour elasticity.

## Results

First, the Pearson’s correlation between the tumour volume and the tumour elastic modulus for all 15 mice prior to drug treatment (i.e. after and including the third scan) was calculated and a moderate positive correlation between elastic modulus and tumour volume (r = 0.37; p = 0.008) was detected. One mouse from a second cohort developed a large scab and a hole in a tumour by the fourth scan and therefore was withdrawn from further results.

**Figure 4:**
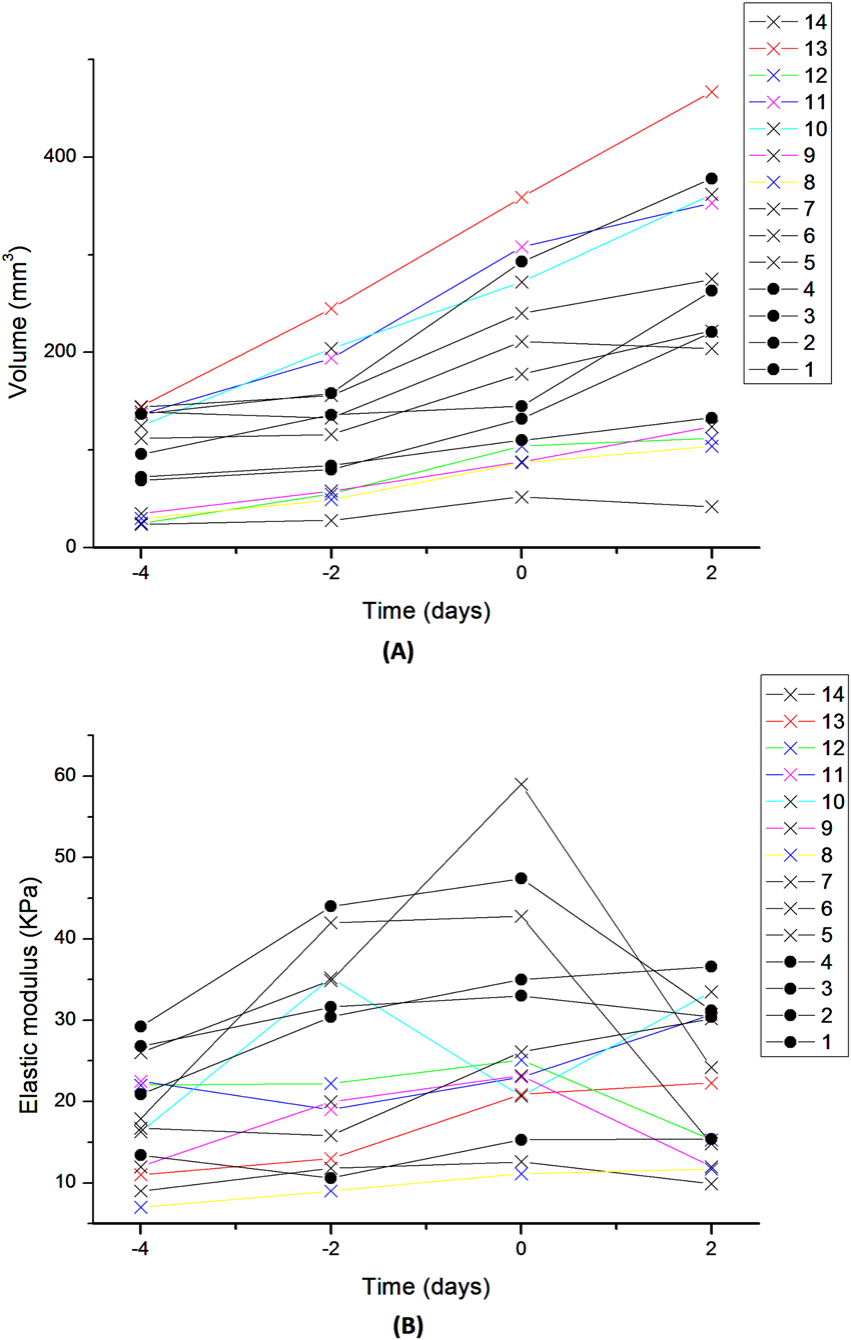
Tumour volume and elastic modulus comparison. Temporal change in tumour volume (**A**) and tumour elasticity (**B**). Day 0 corresponds to the point of treatment. Filled circles  indicate control group and crosses  indicate treated groups.

**Figure 5:**
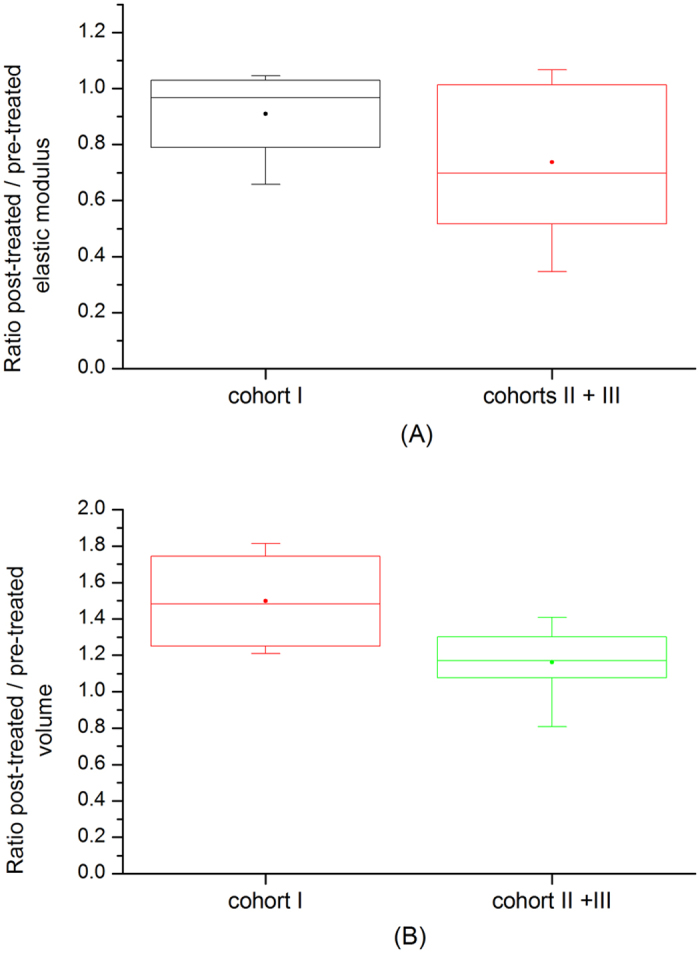
Ratio of post-treatment over pre-treatment tumour volume and elastic (Young’s) modulus. First post-treatment / last pre-treatment ratio of volume (**A**) and elastic modulus (**B**) in control and drug-treated group. The box extends from the 25th to 75th percentiles with min-max whiskers; the mean values are indicated by the dots.

Next, the changes in tumour volume (V) and elastic modulus (E) after treatment were assessed. Here cohort II and cohort III were considered together as one drug-treated group since all conditions were identical up to this point. Figure 4a,b show the values for elastic modulus and volume for all mice. The ratio between first post-treatment (V_2_, E_2_) and last pre-treatment data (V_1_, E_1_) was calculated (Fig. 5). Despite the significant increase in volume in control group as compared with a drug treated group (V_2,control_/V_1,control_ = 1.5 ± 0.29, V_2,drug-treated_/V_1,drug-treated_ = 1.16 ± 0.18, p = 0.02), no significant difference was observed in the change of the elastic modulus suggesting volume is independent of elastic modulus in this model (E_2,control_/E_1,control_ = 0.91 ± 0.18, E_2,drug-treated_/E_1,drug-treated_ = 0.74 ± 0.27, p = 0.27). The elastic modulus did not change in the control group; however in drug-treated mice we observed approximately 25% reduction in elastic modulus, which whilst not statistically significant, highlights a response to the drug. Table 1 summarises the results.

**Table 1:** Detailed results of volume and elastic modulus measurement for each cohort.

	Day -4	Day -2	Day 0	Day 2	Day 4	Day 6	Day 8	Day 10
Volume (mm^2^)								
Cohort I	93.6 (31.3)	114.5 (38.6)	170.0 (83.2)	248.8 (101.8)				
Cohort II	94.0 (56.7)	102.4 (47.0)	160.8 (70.4)	185.8 (70.9)				
Cohort III	106.2 (83.1)	145.2 (97.1)	219.0 (133.6)	267.2 (150.2)	302.2 (157.2)	347.4 (191.1)	378 (136.6)	344
Elastic modulus (kPa)								
Cohort I	25.6 (4.3)	29.2 (13.8)	32.7 (13.2)	28.4 (9.1)				
Cohort II	15.9 (7.1)	24.3 (13.7)	32.4 (18.6)	15.7 (5.1)				
Cohort III	16.2 (6.2)	20.2 (9.4)	21.0 (6.4)	18.7 (6.9)	15.4 (5.0)	14.7 (5.3)	13.7 (4.6)	20

Standard deviation values are given in brackets.

Irinotecan is a topoisomerase I inhibitor which induces DNA damage and leads to cytotoxicity with ensuing apoptosis leading to necrosis within tumours. Given the observed decrease in elasticity we detected using SWE, along with the moderate increase in tumour volume, we reasoned that Irinotecan may be causing tumour necrosis leading to the decrease in tumour elasticity. A significant negative correlation between the degree of necrosis (percentage of tumour) and elastic modulus was observed when the respective data from cohort I and cohort II were analysed (r = −0.73, p = 0.026). A greater negative correlation (r = −0.91, p = 0.03) was also observed in cohort III at the later time point indicating that as necrosis increases following Irinotecan treatment, tumour elasticity also decreases.

**Figure 6:**
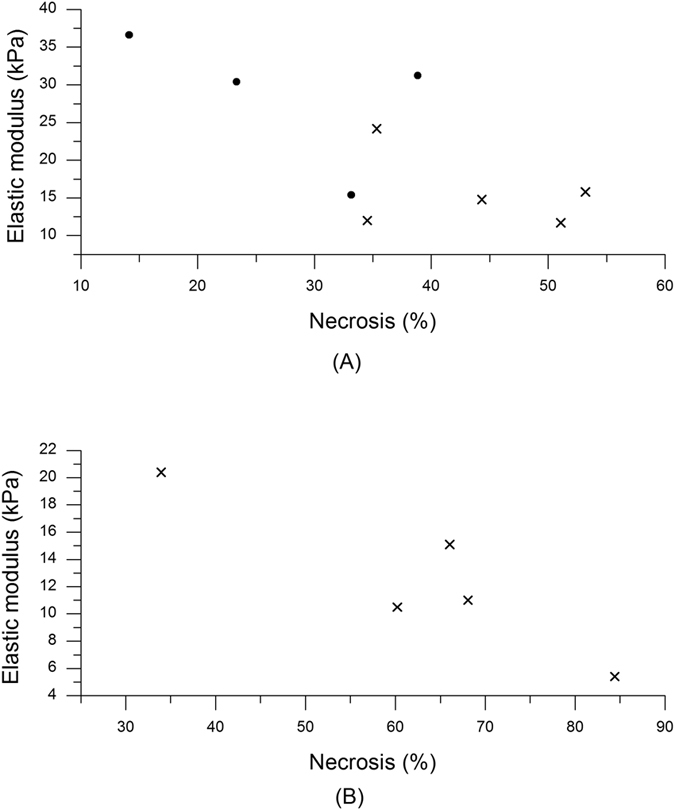
Necrosis as a function of elastic (Young’s) modulus. (**A**) Cohort I () and II (). (**B**) Cohort III.

Figure 6 shows the dependence and Table 2 summarizes these findings.

**Table 2:** Pathological results of all three cohorts.

	Cohort I (day 2)	Cohort II (day 2)	Cohort III (days 6–10)
Last measured elastic modulus (kPa)	28.4 (9.1)	15.7 (5.1)	12.5 (5.6)
Necrosis (%)	27.4 (10.9)	43.7 (8.7); p = 0.04	62.5 (18.3)
Vascularity (%)	0.6 (0.3)	0.4 (0.2)	0.5 (0.2)

The data show the mean value for each group and each feature. Standard deviation values are given in brackets.

In addition, we found that cohort I had a 16% lower degree of necrosis (percentage of total tumour) than it’s matched cohort (II) (p = 0.04).

There was no statistically significant difference in vessel density within the viable areas of the tumour among the groups, suggesting that a loss of vessel density is unlikely to be the cause of tumour necrosis. However, in the control group the vessel density was found to be highly proportional to the tumour volume (r = 0.96; p = 0.038). Only a very small amount of collagen, less than 1%, was detected in the histological analysis, and the staining had inadequate contrast to permit automatic analysis in most of the sections.

**Figure 7:**
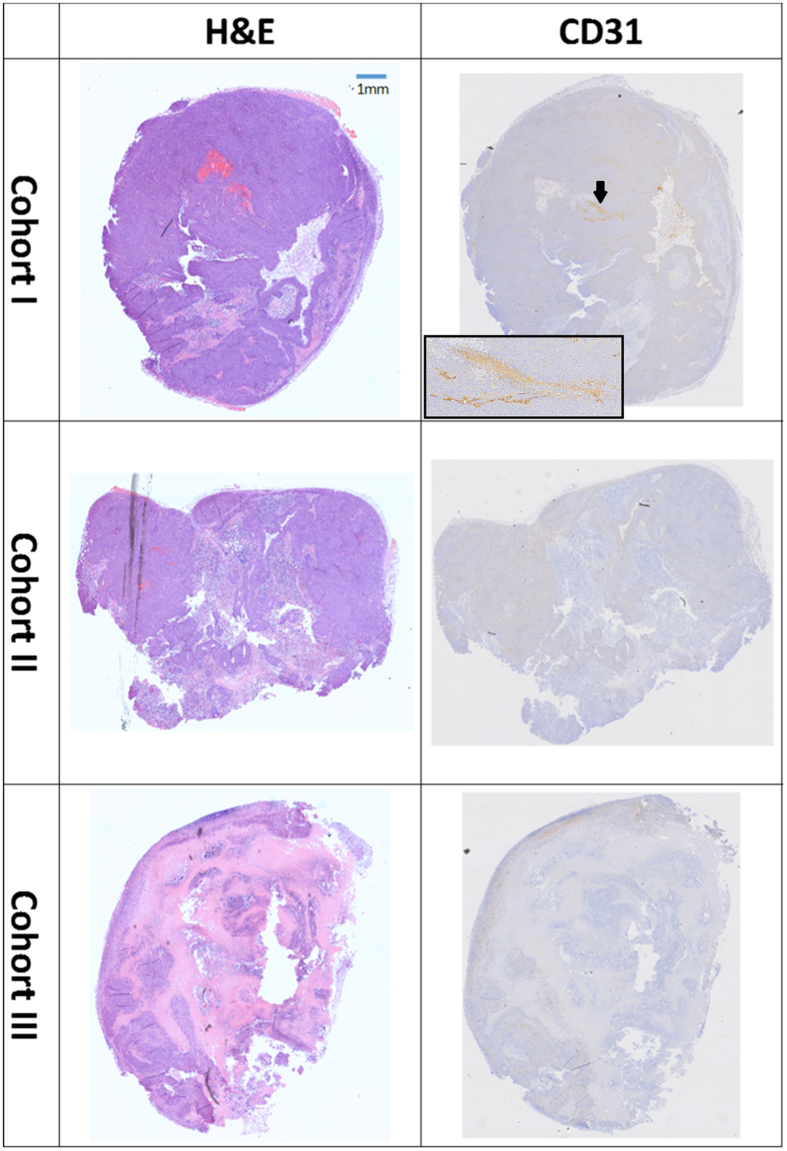
Histological examples of H&E and CD31. In H&E staining (middle column) the purple colour is a viable tissue, pink–necrotic tissue and fat has a white colour. In CD31 (right column) staining the vessels have brown colour as indicated by the black arrow in the upper right image. The inset shows a higher magnification of the vessel.

**Figure 8:**
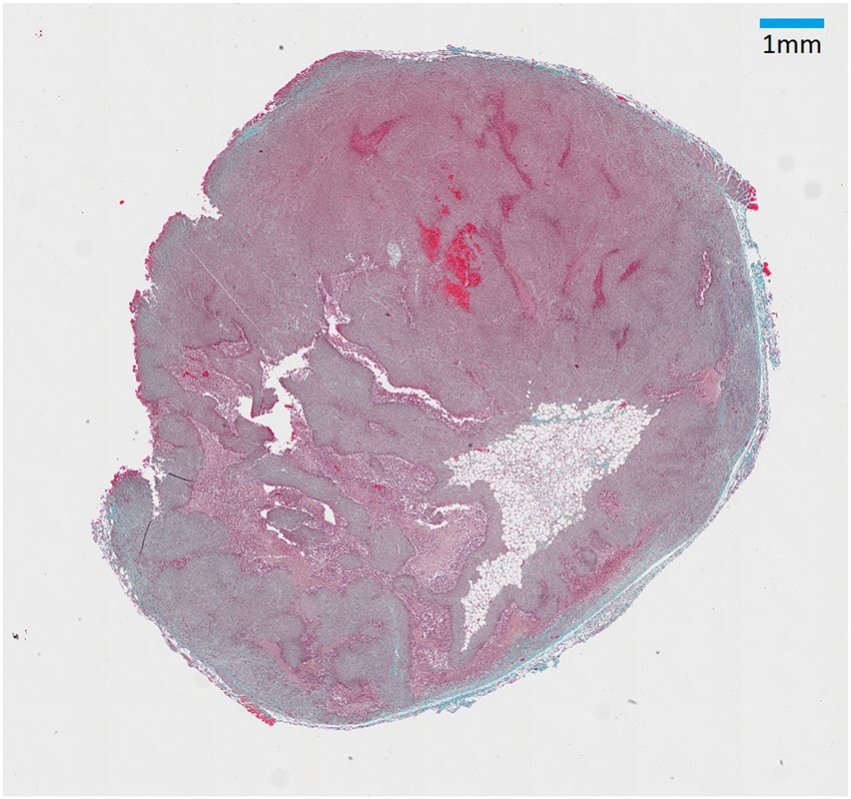
Histological example of Masson’s trichrome. The green-like collagen fibres are discernible, especially close to the periphery, but their extent is small and poor contrast prevents reliable automatic calculation.

Figure 7 shows examples of histological images for H&E and CD31. Figure 8 shows an example of a Masson’s image.

## Discussion

It has been noticed since the dawn of medicine that some diseases are associated with altered tissue stiffness. Among those are several types of cancer, such as breast and prostate, as well as fibrosis of the liver^5^. Elastography is therefore an important tool for tracking pathological processes.

In this study we have shown that US SWE can track changes in elastic modulus in response to cytotoxic therapy. However, we note that the changes in the values of elastic modulus between the drug-treated and the control group are small and did not reach significance in this study. While previous reports in different models with other drugs have observed detectable differences^11^, in the present model the variability appeared too high to robustly draw a conclusion. Prior to Irinotecan treatment, the elastic modulus of tumours increased in all mice concurrently with the tumour volume, which is in line with previously published results^10^, although in the aforementioned study a maximum diameter was used instead of tumour volume.

After the treatment, either with the drug or with saline, tumours continued to grow but the growth rate was slower in the Irinotecan treated group. With regard to the elasticity, the trend was reversed and in all cohorts a decrease in the elastic modulus was observed over time. This is most probably attributed the development of a necrotic core as the necrotic area was found to lie mostly in the centre of the tumour. Thus, while the outer part continued to grow, the core became necrotic. In control cohorts, the lower level of necrosis likely owed to the development of hypoxia (cohort I) indicating a baseline level of tumour necrosis which occurs in developing tumours. However, in Irinotecan treated mice, the increase in necrosis is likely a combination of hypoxia and drug-induced necrosis (cohorts II and III). The same pattern of behaviour was observed by Jugé *et al.*, who used MRE to detect changes in elastic modulus during anti-vascular treatment of Balb-C mice with colon tumours^12^. Although in that work another cell line and another class of drug were used, the common outcome was that necrotic tumours exhibited a lower elastic modulus. This initial increase in elastic modulus is generally attributed to collagen deposition and further post-transitional modifications of extracellular matrix components^18^, along with rapid cellular expansion. In the present study, only a very small amount of collagen was detected. This is, again, in accordance with Jugé *et al.* who also observed a low collagen presence^12^.

In the present study, a statistically significant negative correlation between elastic modulus and necrosis was observed. We show that the percentage of necrosis was significantly higher in both drug-treated cohorts compared to control. This indicates that in addition to the developing hypoxia the drug used in this study causes additional cell death and a breakdown of tumour tissue leading to a decreased elasticity.

Extant literature does not provide a definite answer to the direction of elastic modulus change upon drug treatment. Some studies have reported stiffening^11^, but most have reported softening^19, 20^. In this study, we also observed a decrease in elastic modulus, albeit small; and although we could not differentiate between drug-treated and control groups based on elasticity measurement alone, we could detect pathological changes, as increased necrosis correlated with tissue softening.

We employed an improved method of ROI calculation in the Aixplorer. In the previous studies^10, 11^ only circular ROI provided by the SSI software have been used, while our in-house written software allows free hand drawing of the ROI, thus calculating the elastic modulus only within a chosen area. This improves the accuracy of the calculation as we computed the elastic modulus within the rim of the tumour, regardless of its shape, not necessarily relying on the default circular ROI of the Aixplorer. With improved resolution images and using our method, it would be pertinent to look at elasticity in different regions of the tumour to study heterogeneity in elastic modulus.

We observed a decrease in vascularity, but without statistical significance, and the decrease in CD31 signal is probably related to a smaller viable area within drug-treated cohorts. Whilst our data do not indicate a direct correlation between vascularity and tumour stiffness, further studies would be required to interrogate this. Despite the lack of significant difference in vessel density, in the control cohort it is highly proportional to the tumour volume. This is not surprising, since larger tumours typically require more vascularization^5^.

For future studies a larger number of mice, with tumours excised at different time points, would have allowed a better insight into variation of elastic modulus over time. This is particularly important since measurements in some mice can be difficult owing to highly necrotic tumours, scabs or unresponsive tumours. Secondly, more than two repeated measurements per every scan result might be taken to provide more reliable data. Milkowski *et al.* in their comparison of six commercially available elastography systems acquired ten repeated measurements^21^. To further study the changes in elastic modulus and necrosis one may add another control group that would be comparable to cohort III.

## Conclusions

This study improves our understanding of the dynamics of tumourigenesis with regard to the tumour elasticity. Whether necrosis was caused by drug-treatment or by hypoxia it can be assessed non-invasively by measuring tumour elastic modulus. Elasticity measurements are only one of many available techniques for cancer identification and monitoring of drug efficacy; but as we show here, it provides complimentary information to biopsy and could increase the reliability and accuracy of the results.
